# 3D Algebraic Iterative Reconstruction for Cone-Beam X-Ray Differential Phase-Contrast Computed Tomography

**DOI:** 10.1371/journal.pone.0117502

**Published:** 2015-03-16

**Authors:** Jian Fu, Xinhua Hu, Astrid Velroyen, Martin Bech, Ming Jiang, Franz Pfeiffer

**Affiliations:** 1 Research Center of Digital Radiation Imaging and Biomedical Imaging, Beijing University of Aeronautics and Astronautics, 100191 Beijing, People’s Republic of China; 2 Lehrstuhl für Biomedizinische Physik, Physik-Department and Institut für Medizintechnik, Technische Universität München, 85748 Garching, Germany; 3 Lund University, 22185 Lund, Sweden; 4 School of Mathematical Sciences, Peking University, 100871 Beijing, People’s Republic of China; University of Nebraska Medical Center, UNITED STATES

## Abstract

Due to the potential of compact imaging systems with magnified spatial resolution and contrast, cone-beam x-ray differential phase-contrast computed tomography (DPC-CT) has attracted significant interest. The current proposed FDK reconstruction algorithm with the Hilbert imaginary filter will induce severe cone-beam artifacts when the cone-beam angle becomes large. In this paper, we propose an algebraic iterative reconstruction (AIR) method for cone-beam DPC-CT and report its experiment results. This approach considers the reconstruction process as the optimization of a discrete representation of the object function to satisfy a system of equations that describes the cone-beam DPC-CT imaging modality. Unlike the conventional iterative algorithms for absorption-based CT, it involves the derivative operation to the forward projections of the reconstructed intermediate image to take into account the differential nature of the DPC projections. This method is based on the algebraic reconstruction technique, reconstructs the image ray by ray, and is expected to provide better derivative estimates in iterations. This work comprises a numerical study of the algorithm and its experimental verification using a dataset measured with a three-grating interferometer and a mini-focus x-ray tube source. It is shown that the proposed method can reduce the cone-beam artifacts and performs better than FDK under large cone-beam angles. This algorithm is of interest for future cone-beam DPC-CT applications.

## Introduction

X-ray phase-contrast computed tomography (PC-CT) uses the phase shift that x-rays undergo when passing through matter, rather than their attenuation, as the imaging signal and can provide better image quality in soft-tissue and low atomic number samples. During the last few decades several PC-CT methods have been developed, which are based on crystal interferometer [1–3], free propagation or inline holographic technique [4–7] or diffraction enhancement [8–10]. One of the recent developments is differential PC-CT (DPC-CT), based on a grating interferometer [11–21]. DPC-CT has first been implemented at x-ray synchrotron radiation sources [11–14] and recently transferred to lab-based x-ray tube sources [15–21]. Several experimental studies reported in the literature have demonstrated that DPC-CT offers improved soft-tissue contrast compared to the conventional absorption-contrast CT [16–17, 19, 20].

Most existing DPC-CT approaches are based on three kinds of scanning geometries, i.e., parallel-beam, fan-beam and cone-beam. Favored by the high imaging efficiency and the magnified spatial resolution, cone-beam DPC-CT has attracted significant interest [22–24]. It includes essentially two steps: (i) retrieval of the DPC projections and (ii) phase reconstruction. The first step can be accomplished by using a phase-stepping procedure [12, 14, 15], a reverse projection method [25], or a single-shot Fourier-based phase-extraction method [26]. The second step has so far been solved by using a FDK-type filtered back-projection (FBP) algorithm [22, 23], which is the direct extension of the FDK algorithm commonly used in absorption-based CT [27] by replacing the ramp filter with a Hilbert imaginary filter to take into account the differential nature of DPC projection data. The FDK algorithm is computationally fast and relatively easy to implement. However, as its applications to the absorption-contrast CT, it suffers from the cone-beam artifacts because the necessary and sufficient conditions for accurate CT reconstruction with cone-beam geometry fail [28–31]. There are two major cone-beam artifacts with this approach: loss of contrast intensity and geometrical deterioration along the vertical axis. Both artifacts increase rapidly with the distance to the mid-plane, when the cone-beam angle becomes large.

In the field of conventional x-ray absorption-based CT, iterative reconstruction algorithms have a wide spectrum of proven advantages [32–34], including dose reduction [35, 36], sparse sampling [37, 38], and limited angle tomography [39, 40]. However, there were few studies on iterative reconstruction for DPC-CT in the past. Recently three kinds of iterative reconstruction techniques for fan-beam DPC-CT were proposed, namely, statistical iterative algorithms [32, 41–43], simultaneous algebraic reconstruction technique [44] and algebraic iteration reconstruction [45]. Those studies demonstrated the feasibility of iterative reconstruction for DPC-CT. Although iterative reconstruction exhibits great advantages over FBP-type methods, there is currently no in-depth discussion about the iterative reconstruction technique for cone-beam DPC-CT.

In this work, we have discretized the cone-beam DPC-CT imaging modality as a system of linear equations and developed a 3D algebraic iteration reconstruction (AIR) algorithm for it. This algorithm is based on the Kaczmarz method [46] or the later rediscovered algebraic reconstruction technique (ART) [47] and reconstructs the image ray by ray. Unlike the conventional iterative algorithms for absorption-based CT, it involves the derivative operation in the forward projections to take into account the differential nature of the cone-beam DPC-CT projections. The validity of the proposed AIR method has been demonstrated with numerical simulation and experimental study with a dataset measured at a setup based on a three-grating interferometer and lab-based x-ray mini-focus source. It is shown that the proposed method can reduce the cone-beam artifacts and performs better than FDK under large cone-beam angles.

## Materials and Methods

A three-dimensional object can be described by a complex refractive index distribution *n*(*x*,*y*,*z*) = 1−*δ*(*x*,*y*,*z*)+*iβ*(*x*,*y*,*z*), where *x*, *y* and *z* describe the coordinate system of the sample. In conventional absorption-based CT, the imaginary part *β* is measured by the attenuation of the x-rays transmitted through the specimen. In differential phase contrast imaging, one measures the effect of variations of the real part *δ* by evaluating the tiny refraction angles of x-rays induced by the specimen with a grating interferometer. Correspondingly, under a para-axial approximation, a differential phase-contrast projection can be expressed by

$$\begin{array}{c} \alpha \left(x^{'},z^{'},\theta \right)=\frac{\partial \left(\int_{l(x^{'},y^{'},z^{'})}\delta dl\right)}{\partial x^{'}}, \end{array}$$ (1)

where *x* ^′^ and *z* ^′^ describe the coordinate system of the detector, *θ* the rotation view angle of the object and *l*(*x* ^′^,*y* ^′^,*z* ^′^) the incident ray.

**Fig 1:**
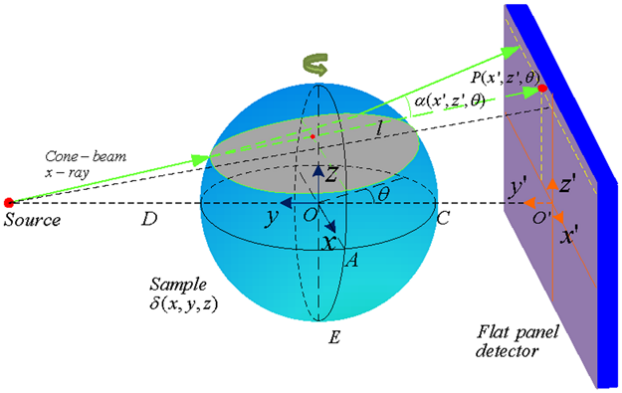
Schematic geometry of x-ray refraction in a medium for cone-beam DPC-CT. (*x* ^′^,*z* ^′^) represents the coordinates of the detector plane. *OAC* is the mid-plane. *D* is the distance from the source to the rotation center *O*. *θ* represents the view angle under which the data was taken. *l* is any incident ray in the three dimensional space under *θ*. *P* is the line integral of *δ* along *l*.

Fig. 1 depicts the scanning geometry of the cone-beam DPC-CT. A sample holder (not shown in the figure) rotates the object over 360^∘^ in a three-grating interferometer during data acquisition. At each view angle, a phase-stepping procedure is performed and provides the DPC projection by Fourier analysis of the recorded moiré fringes by the detector. We are to reconstruct the phase slice images *δ* from the DPC projection.

### 3D Algebraic Iterative Reconstruction Algorithm

To establish the proposed iterative method, we first need to discretize Eq. (1). The reconstructed image is described by a 3D matrix *δ* of size *N*_*image*_ with independent elements *δ*_*j*_, *j* = 1,2,⋯,*N*_*image*_. *δ*_*x*,*y*,*z*_ refers to one voxel for the discretized 3D image *δ* with the voxel index, where

$$\begin{array}{c} j=(z-1)\times (W\times L)+(y-1)\times W+x \end{array}$$ (2)

and *x* = 1,2,⋯,*W*;*y* = 1,2,⋯,*L*;*z* = 1,2,⋯,*H*. Integers *L*, *W* and *H* are, respectively, the length, width and height of the 3D image, which has a total number of voxels *N*_*image*_ = *W*×*L*×*H*.

The phase-contrast projection ∫_*l*_ *δdl* in Eq. (1) is defined as the forward projection and represented by a 3D matrix *P* with size *N*_*prj*_. Each projection is referred to as *P*_*i*_ or *P*_*a*,*b*,*t*_, where

$$\begin{array}{c} i=(t-1)\times (A\times B)+(b-1)\times A+a \end{array}$$ (3)

and *t* = 1,2,⋯,*T*;*a* = 1,2,⋯,*A*;*b* = 1,2,⋯,*B*. Integers *A*, *B* and *T* are, respectively, the columns and rows of the flat panel detector and the number of view angles. The total number of measurements is *N*_*prj*_ = *A*×*B*×*T*.

With the above notations, let *M*_*ij*_ be the weight for the contribution of *j*-th voxel to *i*-th ray in the phase-contrast projection ∫_*l*(*x*^′^,*y*^′^,*z*^′^)_ *δdl*, and *M* be the *N*_*prj*_×*N*_*image*_ matrix. Then we have the following discrete-to-discrete linear transform from the phase image *δ* to the phase-contrast projections *P*

$$\begin{array}{c} P=M\delta . \end{array}$$ (4)

The weights *M*_*ij*_ are computed by calculating the intersection length of the *i*-th ray through the *j*-th voxel. The phase-contrast projection along the *i*-th ray can be expressed by $P_{i}=\sum_{j=1}^{N_{image}}M_{ij}\delta_{j},\;i=1,2,\cdots ,N_{prj}$.

The DPC projections in Eq. (1) are represented by a 3D matrix *α* with size *N*_*prj*_. Each DPC projection *α*_*i*_ = *α*_*a*,*b*,*t*_ at the location (*a*,*b*,*t*) can then be represented by utilizing finite differences of the phase-contrast projections *P*. In this work, we choose to approximate the DPC projections *α* by the phase-contrast projections *P* with the central finite difference. Specifically, for *i* = 1,⋯,*N*_*prj*_,

$$\begin{array}{c} \alpha_{i}=\frac{\left(P_{a+1,b,t}-P_{a-1,b,t}\right)}{2} \end{array}$$ (5)

with

$$\begin{array}{c} t=\text{floor}\left(\frac{i}{A\times B}\right),\;b=\text{floor}\left(\frac{i-t\times A\times B}{A}\right),\;\text{and}\;a=\text{mod}\left(i-t\times A\times B,A\right), \end{array}$$ (6)

where the function floor(*r*) gives the largest integer less than *r* and the function mod(*r*,*s*) the remainder for the division $\frac{r}{s}$.

Now the imaging system, namely, Eq. (1) is discretized by Eqs. (4) and (5). The problem of DPC-CT is then to reconstruct *δ* in Eq. (4) from the measured cone-beam DPC projections *α* in Eq. (5). Obviously, unlike the absorption-based CT, DPC-CT involves the derivative operation in (5) in the forward projections. Although there are many iterative reconstruction methods for absorption-based CT (Please refer to [33–34, 48, 49] for a review on this topic.), they are not applicable for DPC-CT since the simultaneous or block-iterative schemes involve weighted sums of all or multiple ray projections. They could blur the reconstructed image and deteriorate the accuracy of the derivation operation in (5) and hence the spatial resolution.

Based on the above analysis, we developed the following 3D AIR algorithm for cone-beam DPC-CT

$$\begin{array}{c} \left\{\begin{array}{c} \delta [k,i]=0\;if\;k=1,i=0\\ \delta \left[k,i\right]=\delta \left[k,i-1\right]+d\cdot \frac{M_{i}}{\left|\right|M_{i}{\left|\right|}^{2}}\left(\alpha_{i}-\alpha_{i}^{'}\right),\;otherwise. \end{array}\right. \end{array}$$ (7)

Here, *k* and *i* are non-negative integer. *δ*[*k*,*i*] represents the reconstructed image at the *i*_*th*_ inner iteration within the *k*_*th*_ outer iteration. The ray-by-ray reconstruction is called the inner iteration. Once all rays are used, it will start another iteration, which is called the outer iteration. *δ*[1, 0] is the initial guess of the reconstructed image, which is chosen to be the zero at each voxel. The parameter *d* is the relax coefficient and can be from 0 to 2 in theory from the convergence theory for ART [34, 46, 47]. *M*_*i*_ is the projection matrix for the *i*-th ray and $\alpha_{i}^{\prime }$ is the numerical calculation DPC projection by the derivative operation to the forward phase-contrast projection.

The proposed AIR method is based on the algebraic reconstruction technique [47]. It iterates ray-by-ray to update the image *δ*[*k*,*i*] after comparing the measured DPC projection *α*_*i*_ and the numerical projection $\alpha_{i}^{\prime }$ until convergence. Because the derivative operation in Eq. (5) only needs adjacent rays as few as possible, it is expected to provide better derivative estimates in iterations. The overall strategy of this algorithm considers the reconstruction process as the optimization of a discrete representation of the object function to satisfy a system of equations that describes the cone-beam DPC-CT imaging modality, and iteratively arrives at a solution from its cone-beam DPC projection dataset. In the following we discuss the implementation procedure.

### Implementation Procedure

**Fig 2:**
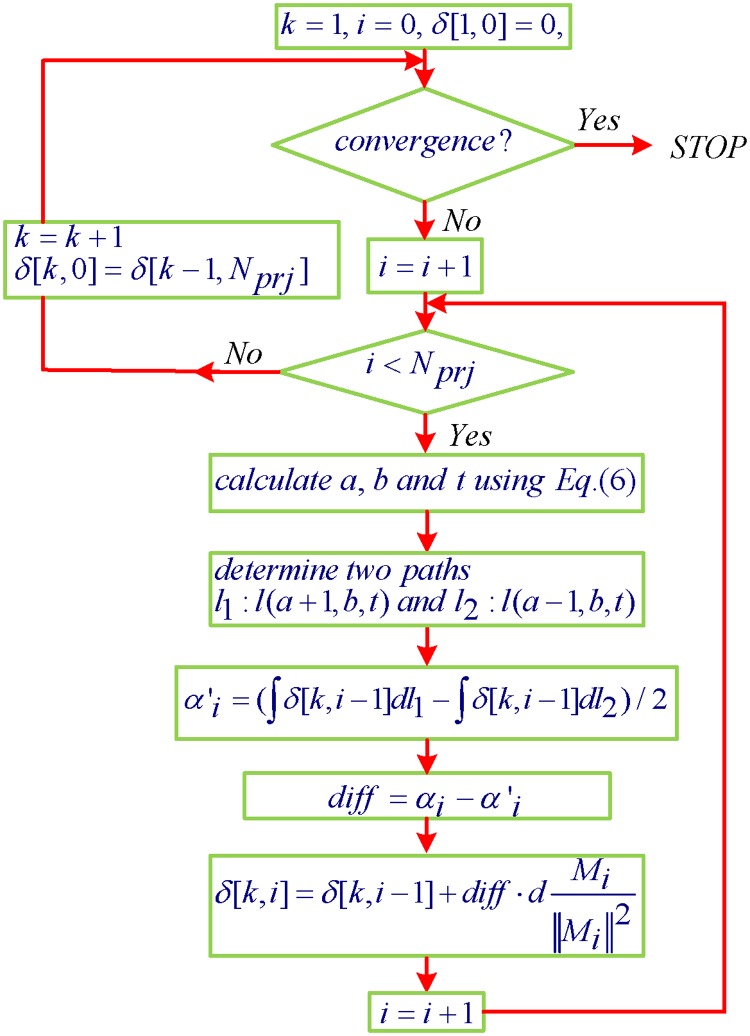
Implementation procedure of 3D algebraic iteration reconstruction for cone-beam DPC-CT.

Having specified the system matrix *M* and data $\vec{\alpha }$, we can describe the implementation of the algorithm in Eqs. (4), (5), (6) and (7). Depicted in Fig. 2, it includes an inner iteration and an outer iteration. This process is labeled in the following way: the outer iteration number is labeled by *k*, and the inner iterations by *i* in their respective loops. The intermediate image during the iterations is noted *δ*[*k*,*i*], indicating the *i*_*th*_ inner iteration within the *k*_*th*_ outer iteration. The successive steps of the algorithm are thus:

(i) Outer iteration initialization.
  $$\begin{array}{c} k=1,\;i=0\;and\;\delta [k,i]=0 \end{array}$$ (8)
(ii) Inner iteration initialization.
  $$\begin{array}{c} i=i+1 \end{array}$$ (9)
(iii) Inner iterative update with the measured data *α* for *i* = 1,…*N*_*prj*_.
  $$\begin{array}{c} \delta \left[k,i\right]=\delta \left[k,i-1\right]+d\cdot \frac{M_{i}}{\left|\right|M_{i}{\left|\right|}^{2}}\left(\alpha_{i}-\alpha_{i}^{'}\right) \end{array}$$ (10)
(iv) Outer iteration convergence check: compute
  $$\begin{array}{c} difference=\delta \left[k,N_{prj}\right]-\delta \left[k,0\right]. \end{array}$$ (11)

If *difference* < *ε*, stop; Otherwise, go to step (ii) to initialize next iteration loop and set

$$\begin{array}{c} k=k+1,i=0,\;and\;\delta \left[k,0\right]=\delta \left[k-1,N_{prj}\right]. \end{array}$$ (12)

Here the parameter *ε* controls the convergence of the algorithm so that the algorithm stops when there is no appreciable change.

## Numerical study

To test the proposed method, numerical simulations were performed. The phantom is adopted from the well-known Defrise phantom [50], which is known as cone-beam killer. It consists of nine discs of diameter 55 mm, vertically separated at different heights of 0, 26.1, 52.4, 78.7 and 105.1 mm (height was measured from the axial mid-plane of whole phantom to the center of each disc). The thickness of each disc was set to 3.0 mm and was attributed refractive index 1×10^−6^. The source-to-object and object-to-detector distances both were set to 1000 mm. The detector has 512×128 pixels with size of each pixel 1.0 mm. The corresponding DPC sinogram was calculated using an algorithm based on the refraction angle equation [1]. The normalized root mean square error (NRMSE) is calculated to evaluated quantitatively the image quality.

**Fig 3:**
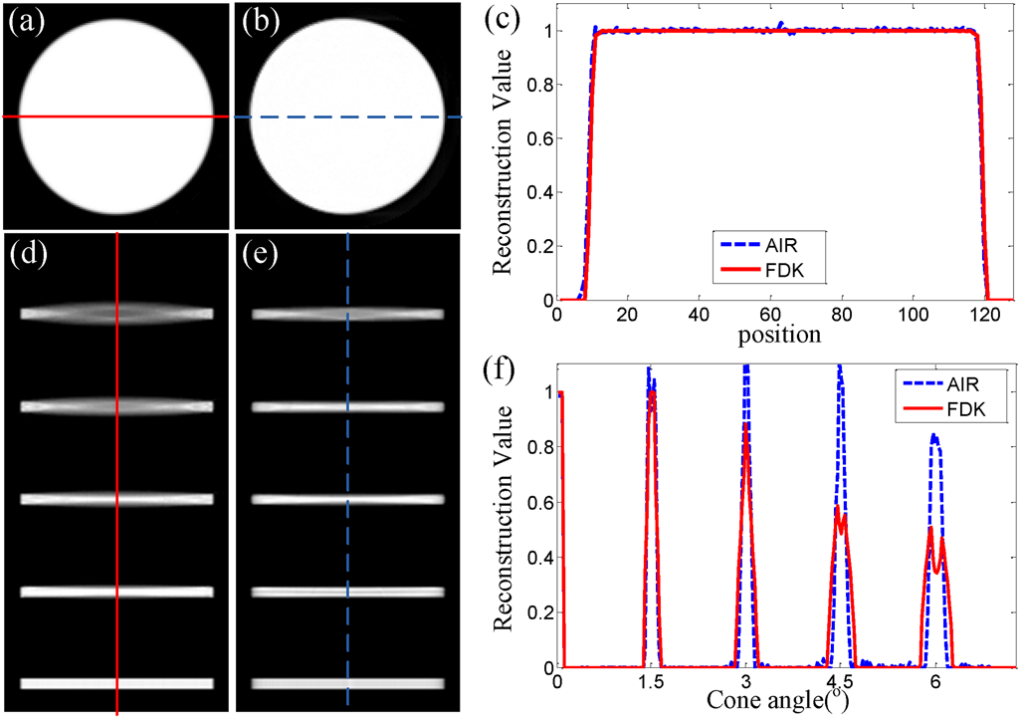
Numerical simulation results. (a) and (b) are the images of the axial mid-plane of the Defrise phantom reconstructed by FDK and AIR algorithms respectively, which correspond to the case with a cone-beam angle of 0^∘^. (d) and (e) are the sagittal slices reconstructed by FDK and AIR algorithms respectively, which have a maximum cone-beam angle of 6^∘^. Starting from the bottom, the central layer of each disc corresponds to a cone angle of 0^∘^, 1.5^∘^, 3.0^∘^, 4.5^∘^ and 6.0^∘^. (c) and (f) present the profiles along the red solid lines and the blue dashed lines in (a), (b) and (d), (e). The display grey scale is set to be [0 1.1]×10^−6^. The relax coefficient is set to be 0.8 and the number of the overall iterations is 10.

Fig. 3 shows the reconstruction results of the Defrise phantom using FDK and AIR algorithms. Figs. 3 (a) and (b) are the images of the axial mid-plane of the Defrise phantom reconstructed by FDK and AIR algorithms respectively, which correspond to the case with a cone-beam angle of 0^∘^. In this case FDK is equivalent to the standard 2D FBP algorithm and can provide exact reconstruction. Obviously in this case AIR method is as accurate as FDK. Fig. 3 (c) further supports this conclusion, which presents the profiles along the red solid line and the blue dashed line in Figs. 3 (a) and (b). It can be also expected by looking at the NRMSE values listed in Table 1.

**Table 1:** The normalized root mean square error in Fig. 3.

	axial slice	sagittal slice
FDK	0.0902	0.6661
AIR	0.0946	0.3165

Figs. 3 (d) and (e) display the sagittal slices of the Defrise phantom reconstructed by FDK and AIR algorithms respectively, which have a maximum cone-beam angle of 6^∘^. In these figures, the cone-beam artifacts are quite visible. In Fig. 3 (d), FDK seems to split every disc into two thinner parts with lower reconstructed values and the extent of the separation of two parts is determined by the cone-beam angle of that height. The profiles presented in Fig. 3 (f) also provides quantitative evidence for this observation. As we can see from Fig. 3 (f), this geometrical distortion becomes more noticeable when the cone-beam angle becomes bigger than 4.5^∘^ and the reconstructed value of the disc drastically drops to nearly half of the theoretical value. In contrast to this, much less artifacts are observed in the image reconstructed by AIR presented in Fig. 3 (e). They are quite consistent with the phantom where even the cone-beam angle reaches 4.5^∘^. Actually the corresponding profile in Fig. 3 (f) demonstrates that the reconstructed values still retain 80% when the cone-beam angle reaches 6^∘^. Furthermore, Listed in table 1, the NRMSE value for AIR sagittal slice is 0.3165 and 52% smaller than the one for FDK sagittal slice. These results support that AIR performs better than FDK when the cone-beam angle increases.

## Experiments

The experimental dataset that was measured to test the reconstruction algorithms was recorded at a CT setup for differential phase-contrast imaging, based on a three-grating interferometer and a mini-focus x-ray tube source installed in a compact gantry at the Technische Universität München (Munich, Germany). The sample was a phantom consisting of a small plastic tube filled with balls of different sizes and different plastic materials.

The experimental setup consisted of a tungsten x-ray source (RTW, MCBM 65B-50 W) with a focal spot size of approximately 50*μm* in diameter. The detector was a flat-panel sensor (Hamamatsu C9312SK-06) with 2496×2304 pixels with 50 *μ*m square pixel size. The gratings of the Talbot-Lau interferometer were fabricated by the company *microworks*, with grating parameters optimized for a design energy of 23 keV. The heights and periods of the grating structures were: 35 *μ*m and 10*μ*m for the source grating *G*0, 40 *μ*m and 3.2 *μ*m for the phase grating *G*1, and 25 *μ*m and 4.8 *μ*m for the analyzer grating *G*2, respectively. The source grating was placed 31 mm from the x-ray tube. The distance between *G*0 and *G*1 was 300 mm, whereas *G*1 and *G*2 were 145 mm apart, corresponding to the first fractional Talbot distance. The source-to-sample and sample-to-detector distances were approximately 270 mm and 200 mm, respectively. A central area of 560×1110 pixels of the detector formed the cone-beam DPC-CT geometrical configuration together with the interferometer and the x-ray generator. The axial cone-beam angle was about 3.4^∘^ and the sagittal cone-beam angle 6.8^∘^.

**Fig 4:**
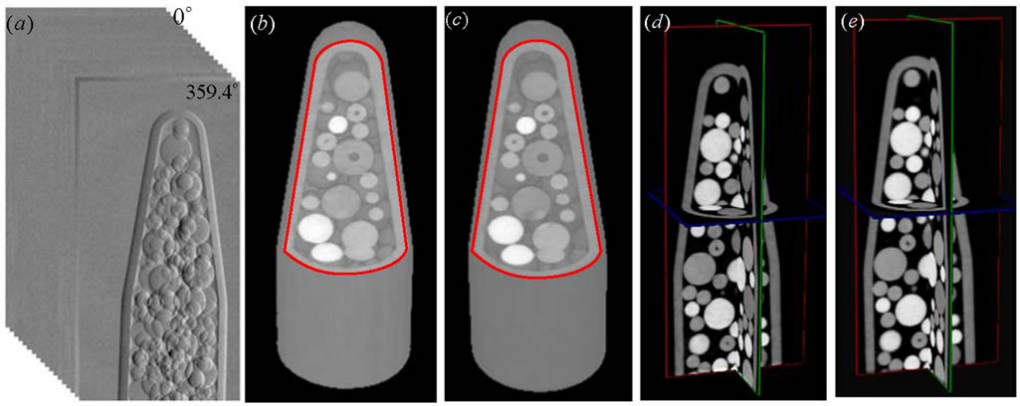
3D reconstruction results of the sample. (a) displays a stack of 2D differential phase contrast projections retrieved from the recorded moiré fringe images by detector. (b),(c) and (d), (e) present the 3D volume rendering and the 3D orthogonal views of the results respectively. (b), (d) and (c), (e) correspond to the results reconstructed by AIR and FDK algorithms respectively. We cut some part of the sample, which is indicated by the red solid curves, to show the internal structure. The relax coefficient is set to be 0.8 and the number of the overall iteration is 10.

The x-ray source was operated at 40 kV and 743 *μ*A. The experimental dataset was acquired by taking 600 projections over 360^∘^ rotation, with 6 phase steps per projection and 5 seconds exposure per phase step. The retrieved DPC projections from all the view angles provide the complete experimental dataset with a size of 560×1110×600 pixels and some of them are displayed in Fig. 4 (a).

To give an impression of the entire reconstruction from the experimental cone-beam DPC data, the 3D volume rendering and the 3D orthogonal views of the results of the sample reconstructed by AIR and FDK algorithms are firstly presented in Fig. 4. From these images, we can find that there exists some internal structure difference in the results, even though they seem to be almost the same.

**Fig 5:**
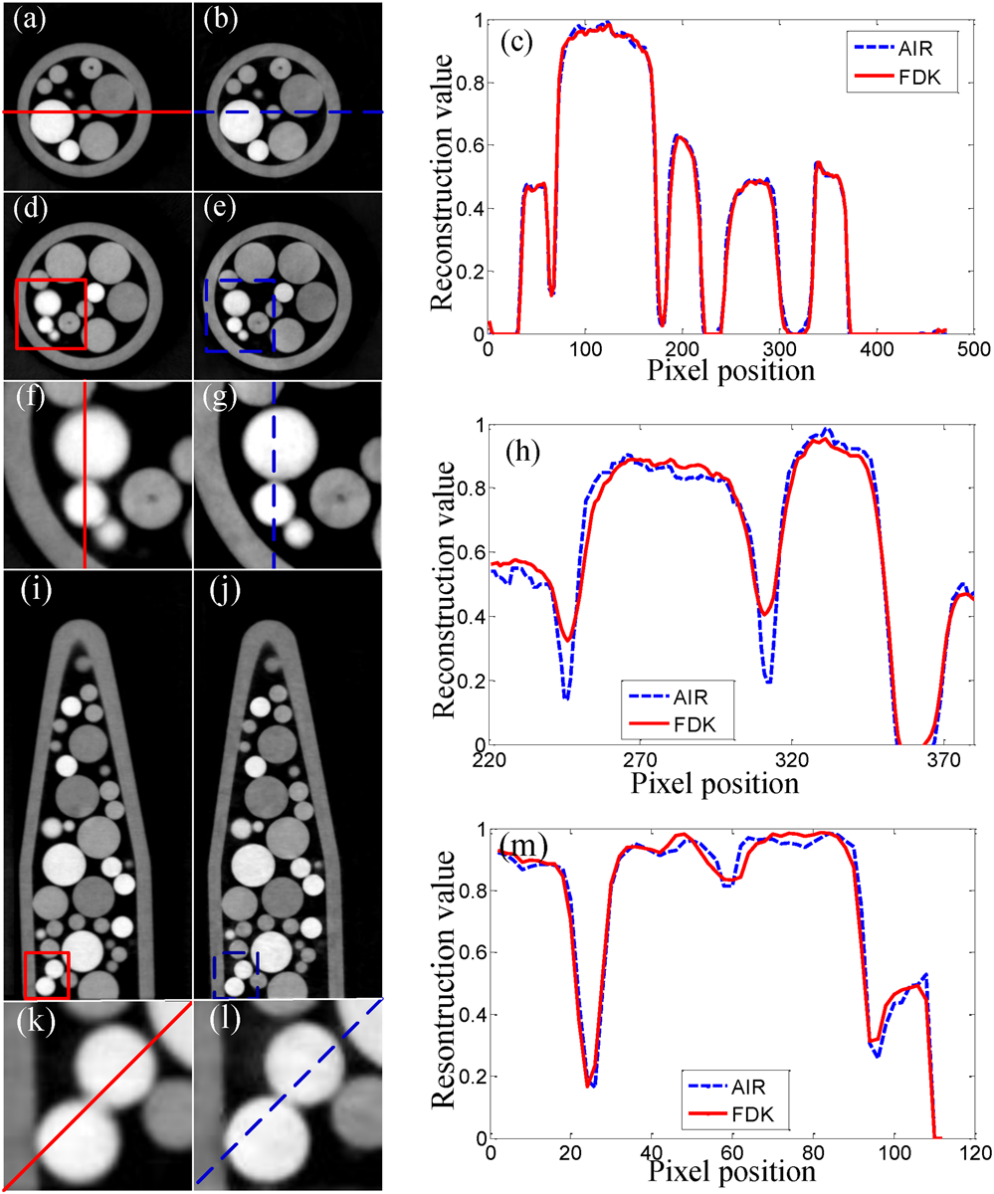
Comparison of experimental reconstruction results in three typical planes. (a), (b) and (c) correspond to the axial mid-plane, (d), (e), (f), (d) and (h) to the axial plane with a cone-beam angle of 2.5 ^∘^ and (i), (j), (k), (l) and (m) to the sagittal mid-plane. (a), (d), (f), (i) and (k) are reconstructed by FDK algorithm. (b), (e), (g), (j) and (l) are reconstructed by AIR algorithm. (f) and (g) are the regions of interest indicated by the red solid rectangle and the blue dashed rectangle in (d) and (e). (k) and (l) are the regions of interest indicated by the red solid rectangle and the blue dashed rectangle in (i) and (j). (c) presents the profiles along the red solid line and the blue dashed line in (a) and (b), (h) in (f) and (g) and (m) in (k) and (l). The reconstructed value is scaled to [0, 1].

For more clarity, the 2D slices in three typical planes (the axial mid-plane, the axial plane with a cone-beam angle of 2.5 ^∘^ and the sagittal mid-plane) are selected and presented in Fig. 5. Figs. 5 (a) and (b) are the slices of the axial mid-plane of the sample reconstructed by FDK and AIR respectively. Fig. 5 (c) depicts the profiles along the red solid line and the blue dashed line in Figs. 5 (a) and (b). As can be seen from Fig. 5 (c), the two profiles coincide well with each other. Obviously both AIR and FDK yield an accurate reconstruction results and achieve comparable image quality at the axial mid-plane.

However, the situation at the axial plane with a cone-beam angle of 2.5 ^∘^ is totally different. Figs. 5 (d) and (e) are the slices at this plane reconstructed by FDK and AIR respectively. Figs. 5 (f) and (g) are the regions of interest in Figs. 5 (d) and (e) indicated by the red solid rectangle and the blue dashed rectangle. The difference between Figs. 5 (f) and (g) is quite visible. The circles in Fig. 5 (f) are more blurred and distorted than in Fig. 5 (g). Some of them are even overlapping with each other on their edges. The profiles presented in Fig. 5 (h), along the red solid line and the blue dashed line in Figs. 5 (f) and (g), also show that FDK produces a serious drop of contrast intensity. AIR undoubtedly performs better than FDK at this plane.

The results at the sagittal mid-plane provides another case to demonstrate the excellent performance of the AIR algorithm. Figs. 5 (i) and (j) are the slices at the sagittal mid-plane reconstructed by FDK and AIR respectively. Figs. 5 (k) and (l) are the regions of interest in Figs. 5 (i) and (j) indicated by the red solid rectangle and the blue dashed rectangle. Like the situation at the axial plane with a cone-beam angle of 2.5 ^∘^, the internal details of the sample in Fig. 5 (k) are more blurred and distorted than in Fig. 5 (l). The profiles presented in Fig. 5 (m), along the red solid line and the blue dashed line in Figs. 5 (k) and (l), further support this observation. It is clear that AIR exhibits better performance than FDK when the cone-beam angle increases.

## Conclusion and Discussion

In summary, we have established an algebraic iterative reconstruction (AIR) algorithm for cone-beam DPC-CT and demonstrated its validity and performance both numerically with simulation and experimentally with real data. This method is based on the well-known algebraic reconstruction technique and tailored to the cone-beam DPC-CT. It is shown that the proposed method can reduce the cone-beam artifacts and performs better than FDK under large cone-beam angles.

It is of practical significance to accelerate the AIR algorithm. In our simulation and experiment, the implementation is with Microsoft Visual C++6.0 in a laptop (LENOVO ThinkPad T400). The laptop is assembled with a Intel Core 2 Duo CPU P8700 at 2.53GHz and a RAM memory of 3 GB. For our experimental reconstruction, it takes about 12 hours to converge. However, this is not an issue since there exist algorithm and hardware (graphics processing unit) to accelerate the algebraic reconstruction technique [51, 52]. The discretization can also be improved with other basis rather the voxel configuration in this work [42]. Future work should be done to improve the reconstruction speed of this algorithm with these techniques toward DPC-CT imaging applications.
